# Supplementary material for: From sequence to enzyme mechanism using multi-label machine learning
Source: BMC Bioinformatics. 2014 May 19;15:150. doi: 10.1186/1471-2105-15-150 (PMC4229970; doi:10.1186/1471-2105-15-150)
Supplement: Additional file 2 — Java code of ml2db. Additional file ml2db_code.tar.gz contains the Java source code to run the multi-label machine learning experiments and save the results to database. The code’s Javadoc is included. [file 1471-2105-15-150-S2.zip › additional file 2/ml2db/ml2db/doc/mulan/classifier/class-use/InvalidDataException.html]

Uses of Class mulan.classifier.InvalidDataException


JavaScript is disabled on your browser.


- Overview
- Package
- Class
- Use
- Tree
- Deprecated
- Index
- Help

- Prev
- Next

- Frames
- No Frames

- All Classes

## Uses of Class mulan.classifier.InvalidDataException

- Packages that use InvalidDataException

  | Package | Description |
  | --- | --- |
  |  |  |
  | --- | --- |
  | mulan.classifier |  |
  | mulan.classifier.neural |  |
- - ### Uses of InvalidDataException in mulan.classifier

    Methods in mulan.classifier that throw InvalidDataException

    | Modifier and Type | Method and Description |
    | --- | --- |
    |  |  |
    | --- | --- |
    | `void` | MultiLabelLearner.`build(MultiLabelInstances instances)` Builds the learner model from specified `MultiLabelInstances` data. |
    | `MultiLabelOutput` | MultiLabelLearnerBase.`makePrediction(weka.core.Instance instance)` |
    | `MultiLabelOutput` | MultiLabelLearner.`makePrediction(weka.core.Instance instance)` Returns the prediction of the learner for a given input `Instance`. |
  - ### Uses of InvalidDataException in mulan.classifier.neural

    Methods in mulan.classifier.neural that throw InvalidDataException

    | Modifier and Type | Method and Description |
    | --- | --- |
    |  |  |
    | --- | --- |
    | `MultiLabelOutput` | MMPLearner.`makePredictionInternal(weka.core.Instance instance)` |
    | `MultiLabelOutput` | BPMLL.`makePredictionInternal(weka.core.Instance instance)` |

- Overview
- Package
- Class
- Use
- Tree
- Deprecated
- Index
- Help

- Prev
- Next

- Frames
- No Frames

- All Classes
